# Low-field magnetic resonance imaging or combined ultrasonography and anti-cyclic citrullinated peptide antibody improve correct classification of individuals as established rheumatoid arthritis: results of a population-based, cross-sectional study

**DOI:** 10.1186/1471-2474-15-268

**Published:** 2014-08-07

**Authors:** Jens K Pedersen, Tove Lorenzen, Bo Ejbjerg, Marcin Szkudlarek, Anne Voss, Mikkel Østergaard, Anders J Svendsen, Lis S Andersen, Kim Hørslev-Petersen

**Affiliations:** 1 King Christian 10th Hospital for Rheumatic Diseases, South Jutland Hospital, Toldbodgade 3, 6300 Graasten, Denmark; 2 Department of Rheumatology, Odense University Hospital, Odense, Denmark; 3 Department of Rheumatology, Regional Hospital Slagelse, Slagelse, Denmark; 4 Department of Rheumatology, University of Copenhagen Hospital at Køge, Køge, Denmark; 5 Copenhagen Center for Arthritis Research, Center for Rheumatology and Spinal Diseases, Glostrup Hospital, Glostrup, Denmark; 6 Department of Clinical Medicine, Faculty of Health and Medical Sciences, University of Copenhagen, Copenhagen, Denmark; 7 Epidemiology, Institute of Public Health, University of Southern Denmark, Odense, Denmark; 8 Institute of Regional Health Services Research, University of Southern Denmark, Odense, Denmark

**Keywords:** Sensitivity and specificity, Ultrasonography, Magnetic resonance imaging, Rheumatoid arthritis, Epidemiology

## Abstract

**Background:**

The aim of the present study was to evaluate the accuracy of two approaches using magnetic resonance imaging (MRI) or combined ultrasonography (US) and anti-cyclic citrullinated peptide antibody (ACPA) for diagnosis and classification of individuals with established rheumatoid arthritis (RA).

**Methods:**

In 53 individuals from a population-based, cross-sectional study, historic fulfilment of the American College of Rheumatology (ACR) 1987 criteria (“classification”) or RA diagnosed by a rheumatologist (“diagnosis”) were used as standard references. The sensitivity, specificity and Area under Curve for Receiver Operating Characteristics curves (ROC-area: (sensitivity + specificity)/2) were calculated for “current fulfilment of the ACR 1987 criteria” (list format), “adapted ACR 1987 criteria” (list format, substituting IgM rheumatoid factor with ACPA and clinical joint swelling and erosions on radiography with synovitis and erosions detected by US on a semi-quantitative scale), and RA MRI scoring System (RAMRIS) scores on low-field MRI in the unilateral hand.

**Results:**

For the ACR 1987 criteria the ROC-area was 75% (sensitivity/specificity = 50%/100%) (with “classification” as standard reference) and 69% (44%/94%) (with “diagnosis” as standard reference), while for the adapted ACR 1987 criteria it was 86% (75%/97%) (classification) and 82% (72%/91%) (diagnosis). For RAMRIS synovitis score in metacarpophalangeal (MCP) joints only (cut-off ≥5), the ROC-area (sensitivity/specificity) was 78% (62%/94%) (classification) and 85% (69%/100%) (diagnosis), while for the total synovitis score of MCP joints plus wrist (cut-off ≥10) it was 78% (62%/94%) (both classification and diagnosis).

**Conclusions:**

Compared with the ACR 1987 criteria, low-field MRI alone or adapted criteria incorporating US and ACPA increased the correct classification and diagnosis of RA.

## Background

Rheumatoid arthritis (RA) is an autoimmune and often incapacitating inflammatory disease primarily affecting synovial joints. The treatment possibilities for patients with RA have improved recently with the emergence of several new disease-modifying antirheumatic drugs (DMARDs) and a focus on early intervention and tight inflammatory control [1]. Over time, a decrease in the numbers of swollen and tender joints, joint damage, disease activity, disability [2,3], and rates for most types of interventions of orthopaedic surgery have also been observed [4-6].

As a consequence, however, it may be increasingly difficult to ascertain individuals with RA in cross-sectional studies aiming to estimate disease occurrence or to verify RA in patients with established disease in clinical settings. In this situation sensitive imaging techniques and specific second generation anti-cyclic citrullinated peptide antibody (ACPA) [7,8] may perhaps provide relevant information.

In patients with arthritis, grey-scale (GS) ultrasonography (US) is more sensitive than clinical examination for detecting synovitis [9,10] and more sensitive than conventional radiography for detecting bone erosions [10,11]. Power Doppler (PD) has been introduced for the assessment of synovitis and may provide additional information [12,13].

There is growing evidence on the performance of low-field, dedicated extremity magnetic resonance imaging (MRI) units for detecting joint inflammation and damage in RA. Low-field MRI is more sensitive than conventional radiography for detecting bone erosions [14] and with high-field MRI as standard reference the sensitivity and specificity for detecting erosions and synovitis is high [15]. In the detection of bone marrow oedema specificity is high but sensitivity only moderate [15,16].

In the field of US and MRI, different working groups have developed standardized and reproducible assessment techniques. For MRI, a semi-quantitative scoring system (RA MRI scoring System, RAMRIS) and a core set of basic MRI sequences have been published [17]. In US, standards for modes of acquisition [18] and definitions of joint pathologies have been proposed [19].

The aim of the present cross-sectional study was to compare the accuracy of two new approaches using either low-field MRI or combined US and ACPA with the American College of Rheumatology (ACR) 1987 classification criteria [20] in individuals with established RA. As gold standard references we used the historical fulfilment of the ACR 1987 criteria and RA diagnosed by a rheumatologist.

## Methods

### Participants

In 2004, a population-based, cross-sectional study of 4,995 randomly selected individuals was conducted in the southern part of Denmark with the aim of describing the point and cumulative prevalence of RA. On the basis of responses to a screening postal questionnaire, telephone interviews and data from local and nationwide registers, 73 individuals from the sample attended a clinical examination. At the examination individuals who had self-reported RA on the screening questionnaire by answering “yes” to the item “Have you or have you ever had RA” were invited to participate in the present study, which was conducted in 2005. Details about the algorithm for inviting individuals to the examination have previously been reported [21].

### Standard references

No true gold standard definition of RA exists, and the most generally used in practice are fulfilment of the ACR 1987 classification criteria. In the present study, data from national and local registers, medical records and questionnaires were collected at hospitals and from general practitioners. All available materials were scrutinized to ascertain individuals in whom the historic fulfilment of the ACR 1987 criteria could be documented (classification) [21], and in the present study this was used as the primary standard reference. To obtain an alternative standard reference the disease status of the participants was evaluated by a rheumatologist (AV) who retrospectively was given access to the results of current investigations (except MRI, ACPA, and US) and documentation from hospitals and general practitioners. The rheumatologist then indicated if the participant had RA or specified an alternative diagnosis.

### Investigations

During interviews, data on symptom duration, morning stiffness, current medications, the Medical Outcomes Study Short Form with 36 items (SF-36) [22] and Health Assessment Questionnaire-Disability Index (HAQ-DI) [23] were collected. Conventional radiography of wrists, hands (posterior-anterior projection) and feet (anterior-posterior projection) were evaluated for Larsen-score [24] by an experienced radiologist, blinded to all other information. According to this method a joint with score ≥2 is erosive. Blood samples were examined for ACPA (EliA IgG, Phadia ImmunoCap, enzyme-linked immunosorbent assay (ELISA), cut-off ≥10 AU/l) [7,8], IgM rheumatoid factor (RF) (ELISA, cut-off >8 IE/ml), erythrocyte sedimentation rate (ESR, mm/hour), and C-reactive protein (CRP, mg/l). In the individuals with RA, disease activity scores were calculated using 28 joints and CRP (DAS28) [25].

### Current 1987 ACR criteria

On the day MRI and US were performed, the participants were examined for the presence of rheumatic nodules, swollen and tender joints (40 joints) by a rheumatologist, blinded to all other information. Fulfilment of the list format of the ACR 1987 criteria on the day of the examination (“current ACR 1987 criteria”) was tested using the following parameters: joint stiffness (≥60 minutes), rheumatic nodules, erosions on radiography (32 joints), RF, and patterns of clinical joint swelling (38 joints).

### US

The participants were examined by US using a General Electric Logiq 9 BT03 ultrasound unit (General Electric, Solingen, Germany) with a 14 Mhz linear active matrix probe with fixed GS and PD settings. US was performed by one of two rheumatologists (TL, LSA), blinded to all other information. Prior to the present study, the examiners performed an evaluation of inter-observer agreement in a pilot study. Based on the semi-quantitative scoring system described by *Szkudlarek et al*, GS synovitis was graded 0-4, US bone erosions 0-3, and PD synovitis 0-3 in the metacarpophalangeal (MCP), metatarsophalangeal (MTP) and proximal interphalangeal (PIP) joints [10,12,26]. In these joints, scores ≥2 were considered to represent definite pathologies. In the wrists, the same features were also evaluated, but only graded 0-1 (0 = absent, 1 = present). Only joints with GS synovitis score ≥1 were examined by PD. The joints were examined in all accessible planes within 15-45 minutes.

### Adapted 1987 ACR criteria

We defined the list format of the *adapted* ACR 1987 criteria using the following parameters: joint stiffness (≥60 minutes), rheumatic nodules, RF substituted with ACPA and clinical joint swelling and erosions on radiography with US synovitis and US erosions (32 joints).

### MRI

On the day the participants were investigated by US, MRI of the non-dominant hand was performed using a 0.2 Tesla Artoscan MRI unit (Esaote Biomedica, Genoa, Italy). The investigation focused on the wrist but, if included in the field of view (FOV), MCP joints 2-5 were also evaluated. Coronal T1-weighted (T1) short tau inversion recovery (STIR) and T1 gradient echo (GE) three dimensional (3D) sequences were performed before and after intravenously injected gadodiamide (0.1 mmol/kg body weight; Omniscan (Amersham Health, Norway)). The following imaging parameters were used: STIR-images: echo time (TE) 18 ms, repetition time (TR) 1100 ms, gap 0.0, slice thickness 3.0 mm, FOV 200 × 200 mm, matrix 256 × 160. T1-GE-3D images: TE 12 ms, TR 30 ms, FOV 140 × 140 × 80 mm, matrix 192 × 160 × 80. The images were evaluated according to RAMRIS [17] by one experienced reader (BE) [27,28]. The RAMRIS scores were assessed for synovitis (possible range for wrist and MCP joints 2-5: 0-21), bone oedema (0-69), bone erosion (0-230), and for the present study a composite score was calculated comprising all three joint pathologies.

### Statistics

Comparisons between groups were made using χ^2^ for binary and Mann-Whitney U tests for continuous variables (level of significance: 0.05; two-sided). The accuracy was evaluated using sensitivity, specificity and Area under Curve (AUC) for Receiver Operating Characteristics (ROC) curves. For specific cut-offs on a scale the ROC-area was calculated as (sensitivity + specificity)/2. The AUC for ROC curves and the ROC-area estimate the correct classification of individuals by the index test. For the RAMRIS scales areas under ROC curves were compared using nonparametric statistics for correlated data [29]. The inter-observer agreement was evaluated using unweighted kappa statistics [30]. Statistics were calculated using Stata, version 8.2. (StatCorp, College Station, Texas).

### Ethics

Informed consent was acquired from all participants and the study was approved by the local ethics committee (Den Regionale Videnskabsetiske Komité for Ringkjøbing, Ribe og Sønderjyllands Amt; reference no. 2426-02) and the Danish Data Protection Agency (reference no. 2002-41-2231).

## Results

**Figure 1:**
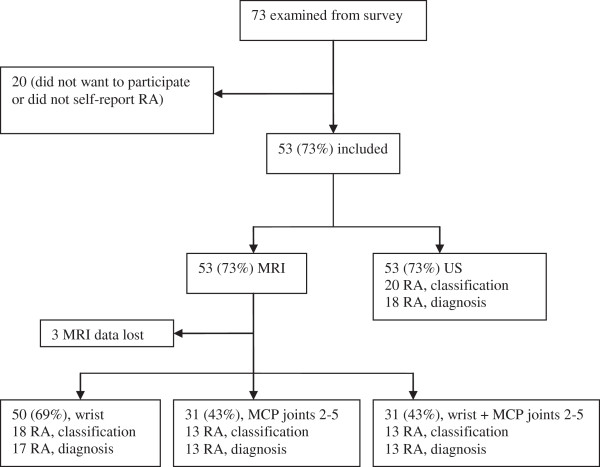
**Numbers of participants in magnetic resonance imaging and ultrasonography analyses.** Historic fulfilment of American College of Rheumatology (ACR) 1987 criteria (classification) or diagnosis with rheumatoid arthritis (RA) by rheumatologist (diagnosis). MCP, metacarpophalangeal. MRI, magnetic resonance imaging; US, ultrasonography.

In the US study, 53 individuals were included; 20 historically fulfilled the ACR 1987 criteria (classification) and 18 were diagnosed as having RA (diagnosis). In three individuals MRI images were damaged during a flood and could not be recovered. In 50 individuals the unilateral wrist was investigated by MRI and in 31 the MCP joints were also included (Figure 1). One individual with RA (according to both standard references) had allergy and was examined by MRI without gadodiamide.

In the group without RA, the individuals were diagnosed with inflammatory and non-inflammatory conditions (Table 1).

**Table 1:** Diagnosis by rheumatologist in the 53 participants

**Diagnosis**	**Numbers (%)**
Rheumatoid arthritis	18 (34)
Unspecified arthritis	1 (2)
Reactive arthritis	3 (6)
Psoriatic arthritis	1 (2)
Juvenile idiopathic arthritis	1 (2)
Arthralgia	16 (30)
Osteoarthritis	9 (17)
Low back pain	1 (2)
Gout	1 (2)
Haemochromatosis	2 (4)

The individuals who historically fulfilled the ACR 1987 criteria (classification) were significantly older, had higher swollen and tender joint counts, ESR and CRP than those who did not. They were also more often erosive on radiography, ACPA and RF positive, currently treated with DMARDs (monotherapy with methotrexate or sulfasalazine in 11, combination therapy in five individuals (including anti-tumor necrosis factor alpha therapy in four)), had higher HAQ-scores, GS synovitis and US erosive joint counts, RAMRIS erosion and oedema scores in the wrist and MCP joints, and synovitis scores in MCP joints. For symptom duration, SF-36 physical component scores, the fraction of females, the numbers of individuals with at least one joint with US erosions, and MRI synovitis scores in the wrist the two groups were not significantly different (Table 2). Four (20%) of the individuals who fulfilled the ACR 1987 criteria had DAS28 <2.6, 14 (70%) had scores ≥3.2, and none had scores ≥5.1.

**Table 2:** Characteristics in 53 participants according to historic fulfilment of the American College of Rheumatology 1987 criteria

	**Rheumatoid arthritis (n = 20)**	**Not rheumatoid arthritis (n = 33)**	**P-value**
Female, n (%)	14 (70)	16 (49)	.178
Age, years	72 (30-79)	59 (31-82)	.008
Symptom duration, years^§^	15 (2-32)	11 (1-53)	.369
Swollen joint count (40 joints)	4.5 (0-10)	0 (0-6)	.000
Tender joint count (40 joints)	10 (0-40)	3 (0-28)	.006
Current treatment with DMARDs, n (%)	16 (80)	0 (0)	.000
CRP, mg/l	5.5 (0-33)	3 (1-14)	.050
ESR, mm/hour	17 (2-53)	5 (1-28)	.000
Positive RF (>8 IE/ml), n (%)	13 (65)	1 (3)	.000
Positive ACPA (≥10 AU/l), n (%)	12 (60)	0 (0)	.000
Positive ACPA and RF, n (%)	11 (55)	0 (0)	.000
SF-36, physical component score*	38 (21-60)	40 (16-57)	.441
HAQ-DI*	0.5 (0-2.375)	0.25 (0-2.125)	.049
DAS28	3.7 (1.8-5.0)	NR	NR
Radiography, erosive (32 joints, ≥1 joint with erosion), n (%)	12 (60)	2 (6)	.000
Radiography, Larsen score	21.5 (0-80)	0 (0-13)	.000
RAMRIS, erosion score (wrist)^#^	4 (0-125)	1 (0-6)	.001
RAMRIS, erosion score (MCP-joints 2-5)^†^	1 (0-8)	0 (0-4)	.009
RAMRIS, bone marrow oedema score (wrist)^#^	2 (0-18)	0 (0-9)	.002
RAMRIS, bone marrow oedema score (MCP joints 2-5)^†^	0 (0-5)	0 (0-2)	.025
RAMRIS, synovitis score (wrist)^#^	4 (1-9)	3 (1-6)	.119
RAMRIS, synovitis score (MCP joints 2-5)^†^	6 (1-8)	3 (0-6)	.017
GS synovitis joint count (30 joints, score ≥2)	4.5 (0-19)	0 (0-6)	.000
GS synovitis joint count (wrists)	1 (0-2)	0 (0-1)	.001
PD joint count (30 joints; score ≥2)	1 (0-14)	0 (0-2)	.000
PD joint count (wrists)	0 (0-2)	0 (0-1)	.001
US erosive joint count (30 joints, score ≥2)	10.5 (0-23)	1 (0-10)	.000
US erosive joint count (wrists)	0.5 (0-2)	0 (0-1)	.000
US erosive (32 joints; ≥1 joint with erosion), n (%)	19 (95)	31 (94)	.871

Values are median (range), unless otherwise stated. Mann-Whitney U test for continuous variables; χ^2^ test for proportions. §Rheumatoid arthritis, n = 19; not rheumatoid arthritis, n = 32. *Not rheumatoid arthritis, n = 32. ^#^Rheumatoid arthritis, n = 18, not rheumatoid arthritis n = 32. ^†^Rheumatoid arthritis, n = 13; not rheumatoid arthritis, n = 18. ACPA, anti-cyclic citrullinated peptide antibody; CRP, C-reactive protein; DAS28, disease activity score in 28 joints using CRP; DMARDs, disease-modifying antirheumatic drugs; ESR, erythrocyte sedimentation rate; GS, grey-scale; HAQ-DI, Health Assessment Questionnaire-Disability Index; MCP, metacarpophalangeal; NR, not relevant; PD, Power Doppler; RAMRIS, Rheumatoid Arthritis Magnetic Resonance Imaging scoring System; RF, IgM rheumatoid factor; SF-36, Medical Outcomes Study Short Form (36 items); US, ultrasonography.

### Index test

The sensitivity, specificity and ROC-area of the list format of the ACR 1987 (“current ACR 1987 criteria”) were 50%, 100% and 75% (classification) and 44%, 94% and 69% (diagnosis) (Table 3). For the list format of the *adapted* ACR 1987 criteria with GS synovitis, US erosions and ACPA, the sensitivity, specificity and ROC-area were 75%, 97% and 86% (classification) and 72%, 91% and 82% (diagnosis). If GS synovitis and US erosions were combined with RF (instead of ACPA), the specificity was slightly lower. Combining GS synovitis, erosions on radiography (instead of US erosions) and ACPA, the sensitivity decreased with no increase in specificity. Using PD synovitis in the adapted ACR 1987 criteria, the specificity was 100% but the sensitivity was lower than for the ACR 1987 criteria (Table 3).

**Table 3:** Sensitivity, specificity and area under Receiver Operating Characteristics curve (ROC-area) of index test

	**Standard references**					
	**Historic fulfilment of ACR 1987 criteria**			**Diagnosis by rheumatologist**		
	**Sensitivity**	**Specificity**	**ROC-area**	**Sensitivity**	**Specificity**	**ROC-area**
**ACR 1987 criteria**						
	50 (27-73)	100 (89-100)	75 (64-86)	44 (22-69)	94 (81-99)	69 (57-82)
**Adapted ACR 1987 criteria (GS synovitis, US erosions, ACPA)**						
	75 (51-91)	97 (84-100)	86 (76-96)	72 (47-90)	91 (77-98)	82 (70-93)
**Adapted ACR 1987 criteria (GS synovitis, US erosions, RF)**						
	75 (51-91)	94 (80-99)	84 (74-95)	72 (47-90)	87 (73-97)	80 (68-92)
**Adapted ACR 1987 criteria (GS synovitis, erosions on radiography, ACPA)**						
	60 (36-81)	97 (84-100)	79 (67-91)	56 (31-79)	91 (77-98)	73 (61-86)
**Adapted ACR 1987 criteria (PD synovitis, US erosions, ACPA)**						
	35 (15-59)	100 (89-100)	67 (57-78)	39 (17-64)	100 (90-100)	69 (58-81)
**RAMRIS scale for synovitis**						
MCP joints 2-5 (cut-off ≥5)	62 (32-86)	94 (73-100)	78 (63-93)	69 (39-91)	100 (73-100)	85 (72-98)
Combined wrist and MCP joints 2-5 (cut-off ≥10)	62 (32-86)	94 (73-100)	78 (63-93)	62 (32-86)	94 (73-100)	78 (63-93)

Historic fulfilment of the American College of Rheumatology (ACR) 1987 criteria (classification) or diagnosis with rheumatoid arthritis by rheumatologist (diagnosis) as standard reference, % (95% confidence interval). ACPA, anti-cyclic citrullinated peptide antibody; GS, grey-scale; MCP, metacarpophalangeal; PD, Power Doppler; RAMRIS, Rheumatoid Arthritis Magnetic Resonance Imaging scoring System; RF, IgM rheumatoid factor; US, ultrasonography.

**Figure 2:**
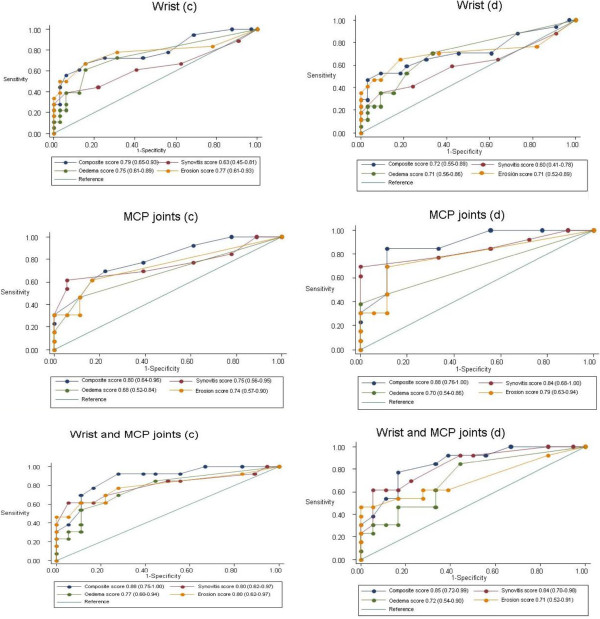
**Areas under Receiver Operating Characteristics curves for Rheumatoid Arthritis Magnetic Resonance Imaging scoring System.** Bone oedema, bone erosion, synovitis and composite scales in unilateral wrist and metacarpophalangeal (MCP) joints 2-5 with historic fulfilment of American College of Rheumatology 1987 criteria (classification, **c**) or diagnosis with rheumatoid arthritis by rheumatologist (diagnosis, **d**) as standard references (95% confidence interval).

Looking at MRI in the MCP joints at cut-off ≥5 for synovitis, the sensitivity, specificity and ROC-area were 62%, 94% and 78% (classification) and 69%, 100% and 85% (diagnosis). For the wrist and MCP joints combined at cut-off ≥10 for synovitis, the sensitivity, specificity and ROC-area were 62%, 94% and 78% (same values for both classification and diagnosis) (Table 3). At no other cut-off for the RAMRIS scales in the wrist, MCP, or MCP and wrist joints combined was the ROC-area higher than for the ACR 1987 criteria (Figure 2).In the wrist, MCP and wrist and MCP joints combined, the AUC for the composite scale was higher than the AUC for the other joint pathologies detected by MRI (Figure 2).

### Pilot study

In the preceding pilot study, a total of 112 joints from four RA patients (seven wrists, 35 MCP, 35 PIP, 35 MTP joints) were evaluated by US. Overall, the kappa values for definite joint pathologies were 0.80 (95% confidence interval (CI): 0.63-0.97) for GS synovitis, 0.75 (95% CI: 0.56-0.94) for PD synovitis, and 0.84 (95% CI: 0.73-0.97) for US erosions, corresponding to good or very good agreement.

## Discussion

The main finding of our study was that the correct classification of individuals with established RA was improved with two new approaches using low-field MRI or combined US and ACPA. Compared with the ACR 1987 criteria (“current ACR 1987 criteria”), the ROC-area increased from 75% to 86% using the adapted ACR 1987 criteria (with US synovitis, US erosions, and ACPA) with the historic fulfilment of the ACR 1987 criteria (classification) as standard reference. Looking solely on MRI synovitis in the MCP joints or wrist and MCP joints combined, the ROC-area increased to 78% (i.e. without incorporating information from clinical, biochemical or other imaging parameters). Although many of the individuals with RA were currently treated with DMARDs, the sensitivity and specificity of the adapted ACR 1987 criteria was close to the accuracy of the ACR 1987 criteria in a recent meta-analysis (sensitivity 79-80%, specificity 90-93%) [31].

In the US pilot study, the inter-observer agreement for definite joint pathologies was high. Originally *Szkudlarek et al* did not include the wrist in their scoring system [10,12,26], but today we could have used a more detailed scale for synovitis in the wrist [32]. Moreover, if all the joints had been examined for PD synovitis, the sensitivity of the adapted ACR 1987 criteria using PD synovitis might have been higher.

The numbers of participants with at least one joint with US erosions was equally high among those who were classified as having RA and those who were not. In previous studies erosions have often been detected in healthy individuals [33] and individuals with arthritides as diagnosed in our study [34-36].

To our knowledge, the accuracy of an approach including US as a criterion for the classification of individuals with RA in a cross-sectional design has not previously been systematically evaluated.

As a single test for RA, ACPA and RF have equal sensitivity but ACPA has higher specificity [37]. In two previous, cross-sectional studies in hospital patients, substituting rheumatic nodules and erosions on radiography with ACPA, the sensitivity of the ACR 1987 criteria increased with a decrease in specificity [38,39]. Adding ACPA as a criterion to the ACR 1987 criteria, the sensitivity improved with little loss in specificity [39]. In our study, most of the individuals with RA were both ACPA and RF positive and the majority of those without RA where ACPA and RF negative. This explains why the accuracy of the adapted ACR 1987 criteria changed only slightly when ACPA was used instead of RF.

Turning to MRI, with the diagnosis as standard reference the ROC-area (cut-off >5) on the RAMRIS scale for synovitis in the MCP joints was 85%. Most of the individuals with RA had moderate disease activity and were treated with methotrexate or sulfasalazine as monotherapy and our results confirm that with MRI it is possible to detect subclinical synovitis [40]. On the other hand, using a low-field MRI unit and examining one individual with RA without contrast agent we probably underestimated the occurrence of bone marrow oedema [15,16] and synovitis [41]. The non-dominant hand was examined by MRI but based on previous reports [42,43], we do not think that this has biased our results.

The AUC for the ROC curves of the composite scale comprising all three joint pathologies defined by RAMRIS was highest in every joint area, indicating that both damage and inflammation contributed with relevant information for the correct classification of the individuals. RA is a polyarticular disease and if more joint areas had been investigated the AUC of the composite scale might have been higher. However, the inclusion of more joint areas will increase the acquisition time and using RAMRIS guidelines requires that contrast medium is administered repeatedly and this would make low-field MRI less patient-friendly. In a previous cross-sectional study of consecutive outpatients diagnosed with RA and healthy individuals, low-field MRI of the bilateral wrist and MCP joints 2-5 was evaluated using a modified version of RAMRIS without contrast agent. The sensitivity and specificity were 65% and 83% for bone oedema (the cut-off was not clearly stated), 70% and 80% for synovitis (score >2) and 55% and 90% for erosions (>5 erosions) [43].

Our study was conducted in 2005 with the ACR 1987 criteria to test. Recently, new classification criteria for RA have been introduced (2010 ACR/European League Against Rheumatism (EULAR) criteria) for the early identification of individuals with arthritis at high risk of developing erosive or persistent disease [44]. The participants did not all have at least one swollen joint at the clinical examination, which is the eligibility criterion for testing with the 2010 ACR/EULAR criteria. Consequently, we did not perform *post hoc* analyses with the new criteria. Neither did we use previously described criteria for the ascertainment of individuals with RA in twin studies [45].

However, after the introduction of the new criteria for RA it seems that a fraction may only fulfil the ACR 1987 criteria [46]. Individuals who fulfil the new criteria may often achieve drug free remission [47] and it is not clear if the new criteria may be applied meaningfully in a retrospective or cumulative way [48]. It therefore still seems relevant to estimate the incidence and prevalence of RA using the 1987 ACR criteria, until more descriptive data with the 2010 ACR/EULAR criteria have been published.

The main weakness of the present study is that the low numbers of participants resulted in rather wide and overlapping confidence intervals for the point estimates of accuracy. Nevertheless, the participants were recruited from a random sample of the general population and we think that the approaches described here may be used in future epidemiological studies conducted with the aim of estimating the prevalence of RA in a selected population.

In our opinion, the high fraction of individuals with non-inflammatory conditions after the diagnostic workup reflects the general methodological problem that in population based studies aiming to describe the prevalence of RA, it is difficult to identify individuals at high risk of having RA [21]. Many of the participants in our study had actually been seen in ambulatory settings and our results may also apply to a hospital outpatient population. However, since the accuracy of an index test may change with the disease severity among the cases and the spectrum of diseases among the non-cases [49], this assumption should be tested in a consecutive series of outpatients.

## Conclusions

In this cross-sectional study the correct classification of individuals with established RA was improved over what was seen for the ACR 1987 criteria by two new approaches either using low-field MRI or a combination of US and ACPA. Our results may apply to cross-sectional epidemiological studies conducted with the aim of estimating the prevalence of RA. Whether they also apply for patients in ambulatory settings has to be confirmed.

### Support

The contrast agent was sponsored by Amersham Health, Norway. The study was funded by the Region of Southern Denmark (formerly the County of South Jutland), Denmark; the Danish Rheumatism Association; the Margarethe Astrid Hedvig Schaufuss Grant; Grocer Hans Christensen’s Memorial Grant. The work by AV was sponsored by an individual grant from the Danish Rheumatism Association (R33-A1836) and the AP Møller Foundation.

## Abbreviations

ACPA: Anti-cyclic citrullinated peptide antibody; ACR: American College of Rheumatology; AUC: Area under curve; CRP: C-reactive protein; DAS28: Disease activity score in 28 joints using CRP; DMARDs: Disease-modifying antirheumatic drugs; ELISA: Enzyme-linked immunosorbent assay; ESR: Erythrocyte sedimentation rate; EULAR: European League Against Rheumatism; FOV: Field of view; GE: Gradient echo; GS: Grey-scale; HAQ-DI: Health Assessment Questionnaire-Disability Index; MCP: Metacarpophalangeal; MRI: Magnetic resonance imaging; MTP: Metatarsophalangeal; NR: Not relevant; PD: Power Doppler; PIP: Proximal interphalangeal; RA: Rheumatoid arthritis; RAMRIS: Rheumatoid Arthritis Magnetic Resonance Imaging scoring System; RF: IgM rheumatoid factor; ROC: Receiver Operating Characteristics; SF-36: Medical Outcomes Study Short Form; STIR: Short-tau inversion recovery; TE: Echo time; TR: Repetition time; T1: T1-weighted; US: Ultrasonography; 3D: Three dimensional.

## Competing interests

JKP, BE, MS, AV, MØ, AJS, LSA, KHP: None declared. TL: Advisory Board member, Roche and Pfizer.

## Authors’ contributions

Conception and design of study: JKP, TL, MS, MØ, AJS, KHP, LSA. Acquisition of data: JKP, TL, BE, AV, LSA, KHP. Data analysis: JKP, TL. Drafting of manuscript: JKP. Interpretation of data, and revision of manuscript: all authors. All authors read and approved the final manuscript.

## Pre-publication history

The pre-publication history for this paper can be accessed here:

http://www.biomedcentral.com/1471-2474/15/268/prepub
